# Cancer, Retrogenes, and Evolution

**DOI:** 10.3390/life11010072

**Published:** 2021-01-19

**Authors:** Klaudia Staszak, Izabela Makałowska

**Affiliations:** Institute of Human Biology and Evolution, Faculty of Biology, Adam Mickiewicz University in Poznan, 61-614 Poznan, Poland; klaudia.staszak@amu.edu.pl

**Keywords:** retrogenes, retroposition, cancer, tumor evolution, species evolution

## Abstract

This review summarizes the knowledge about retrogenes in the context of cancer and evolution. The retroposition, in which the processed mRNA from parental genes undergoes reverse transcription and the resulting cDNA is integrated back into the genome, results in additional copies of existing genes. Despite the initial misconception, retroposition-derived copies can become functional, and due to their role in the molecular evolution of genomes, they have been named the “seeds of evolution”. It is convincing that retrogenes, as important elements involved in the evolution of species, also take part in the evolution of neoplastic tumors at the cell and species levels. The occurrence of specific “resistance mechanisms” to neoplastic transformation in some species has been noted. This phenomenon has been related to additional gene copies, including retrogenes. In addition, the role of retrogenes in the evolution of tumors has been described. Retrogene expression correlates with the occurrence of specific cancer subtypes, their stages, and their response to therapy. Phylogenetic insights into retrogenes show that most cancer-related retrocopies arose in the lineage of primates, and the number of identified cancer-related retrogenes demonstrates that these duplicates are quite important players in human carcinogenesis.

## 1. Introduction

A large part of the eukaryotic genome contains sequences that result from the activity of transposable elements. Until recently, most of them were considered insignificant. It turned out that transposable elements importantly influenced the evolution of genomes. Identifying the possible functions of “junk DNA” constitutes one of the greatest discoveries in genomic analyses [1,2,3]. The term “junk DNA” also refers to pseudogenes; however, an increasing number of studies support the functionality of many pseudogenes and their role in various human diseases. Findings from studies in cell culture, animal models, and clinical samples confirm the role of pseudogenes in tumorigenesis [4,5,6]. This includes retrocopies that are usually called “processed pseudogenes” or “retropseudogenes” and have been classified as gene copies with no functional significance. Nevertheless, as the quantity of data from high-throughput experiments increases, new functionally important retrocopies are identified, including those associated with various diseases [7,8], especially with many types of cancer [9,10].

The phenomenon of neoplasm is widespread across the evolutionary tree, but its incidence varies between species. Surprisingly, the risk of cancer transformation does not seem to depend strongly on individual species’ body size or life expectancy [11]. Studies on a group of elephants have shown that among them, there is a low rate of tumor transformation compared to other mammals. Elephants have 20 copies of the *TP53* gene, a well-known oncosuppressor, 19 of which are retropseudogenes. The presence of this gene’s additional copies is proposed to be related to an increased apoptotic response in the elephant population [12]. The occurrence of resistance mechanisms to cancer has also been described in other animal species. A good example is the unusual tolerance to hypoxia in naked mole-rats [13].

**Figure 1:**
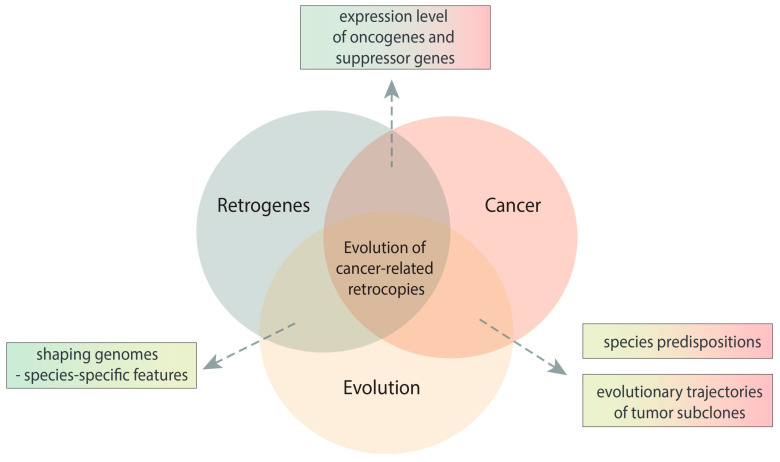
Schematic view of the relationship between retrogenes, cancer, and evolution.

Many studies have described the relationship between the expression level of retrogenes and the incidence of specific neoplasms [14,15], and the connection between these two appears to go beyond this. Retrogenes are known as important evolutionary players that can affect genome diversity [16,17,18,19,20], which is due to, among other things, their much faster evolution than in the case of protein-coding genes. In cancer, there is also a rapid accumulation of mutations and the formation of qualitatively different populations of cancer cells. In addition, it has been demonstrated that internal diversity is the result of natural selection, which shapes the tumor during its development [11]. Natural selection evidently also determines the fate of retrocopies. These connections between retrogenes and cancer gave reasons for an evolutionary-based overview of retrogenes in cancerous contexts (Figure 1).

## 2. Retrocopies and Their Functions

New gene copies may be obtained by polyploidization, irregular crossing over, or DNA- or RNA-mediated duplication [21]. Until recently, only DNA-based duplication was considered to be functionally relevant. However, later studies have revealed that RNA-based duplication provides copies that may play a vital role in the cell [3,22,23,24].

**Figure 2:**
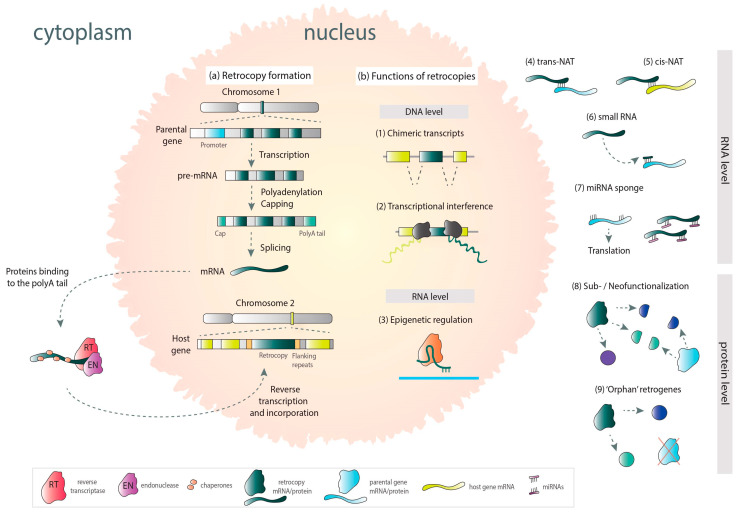
Mechanism of retrocopy formation (**a**) and possible functions in the cell (**b**) (based on mechanisms collectively described by Kubiak et al. [3]).

The formation of retrocopy begins with the transcription of a parental gene. The processed mRNA goes to the cytoplasm, where L1 retrotransposon-derived proteins bind to its polyA tail. The process takes place with the participation of reverse transcriptase, endonuclease, and chaperones. Parental gene’s mRNA anneals to the broken DNA ends, undergoing reverse transcription, and the resulting cDNA is integrated back into the genome in the form of a retrocopy (Figure 2a). Retrocopies are devoid of introns and regulatory elements. They are equipped with a poly-A tail along with flanking repeats. These copies were long considered to be “dead-on-arrival” and classified as transcriptional noise due to their high similarity to the parental genes. Nevertheless, to promote transcription, retrotransposed transcripts can take advantage of adjacent gene regulatory regions and use distant CpG sequences or sometimes even parts of their own sequences. Furthermore, retrocopy insertion into the intron of another gene often leads to the acquisition of the host’s regulatory machinery [22,25].

Retrocopies that acquired transcriptional ability are called retrogenes. In the process of evolution, they may be subject to subfunctionalization and take over some of the parental genes’ functions (Figure 2b). A good example is retrogene *SLC5A3* and the parental gene *SLC5A1*. Proteins encoded by these genes contain solute binding domains, but they differ in activity. Retrocopy-derived protein is a sodium-dependent myo-inositol transporter. In turn, parental-derived proteins participate in the transport of glucose and galactose [24]. Another functional evolution path of retrocopies is neofunctionalization. As a result, they can encode proteins, novel or similar to those encoded by the parental gene, or they can obtain regulatory functions and be involved in transcriptional regulation of parental counterparts or other genes. They can also participate in transcriptional interference, be a source of different small RNAs, or act as miRNA sponges [24,26,27]. Retrogene-derived RNAs can also be involved in epigenetic regulation [28] or function as trans-NATs (natural antisense transcripts) [24,29]. Moreover, it was demonstrated that retrogenes can functionally replace their parental genes [8]. Retrocopies may also contribute to other genes and/or transcripts; they can create chimeric transcripts, act as recombination hot spots [24], or provide a sequence for alternative exons [30].

## 3. Retrogenes and Evolution

It is widely understood that adaptive features, lineage-specific phenotypic traits, are associated with the formation of new genes [22]. Gene duplication is a primary mechanism of new gene formation by providing a substrate for natural selection. The additional copies of ancestral genes are subject to less evolutionary restriction to develop a novel feature [21]. They accumulate mutations faster than protein-coding genes and thus evolve faster [31]. Within the different types of duplication, there are differences in the susceptibility to evolutionary changes. Duplication at the DNA level results in daughter copies with full equipment (core promoters and gene organization). Therefore, these duplicates mostly mirror the protein function and expression pattern of their ancestor [25]. In contrast, analysis of retrogenes has pointed to their significant contribution to molecular evolution as a source of genomic novelties, and they are called “seeds of evolution” [1]. Due to the lack of regulatory elements, transcribed retrocopies must acquire regulatory regions. Thus, retrocopies are probably more predisposed to evolve a novel expression pattern and functional role than copies emerging from segmental duplication. Moreover, retrogenes play a role in gene structure evolution by mediating the decline of introns [22]. Nevertheless, retrocopies may gain introns or additional exons over time. Szcześniak et al. reported two retrogenes, *RNF113B* and *DCAF12*, where introns were created through mutations and the appearance of new splice sites [19]. On the other hand, Vinckenbosch et al. identified 27 intergenic retrogenes that acquired de novo exons [25].

Several studies support the hypothesis that splicing signal conservation constrains the rate of protein evolution [22], it has been suggested that the evolution rate is lower within the exon-intron boundaries and for intron-rich genes [32]. Therefore, splicing constraints impose some limitations on parental gene evolution. However, such constraints should not apply to single exon retrocopies. Interestingly, it has been noted that within the retrocopy sequence, the rate of protein evolution is in fact the strongest within previous splicing junctions in the ancestor gene. Consequently, a more effective adaptation of retrogene-derived protein in comparison with the parental gene’s protein can be speculated as a result of relaxing splicing constraints [22].

Many studies emphasize the role of retrogenes in the differentiation and molecular evolution of genomes [20,24,30] and, as a result, are a source of species-specific features as well as interspecies variation. For example, a retrocopy of the cyclophilin A gene within the owl monkey genome is associated with resistance to HIV [20]. Another example constitutes the rodent-specific retrogene *Rps23r1*, which reduces Alzheimer’s β-amyloid levels and may cause discrepancies between animal model studies and results of clinical trials, for example [33]. Furthermore, the *fgf4* retrogene that is responsible for chondrodysplasia is found only in short-legged dog breeds [17]. Finally, as previously mentioned, the increased number of *TP53* gene retrocopies results in a lower cancer transformation rate in the elephant population [12]. Moreover, retrocopy number variation was also observed across human populations. This includes transcriptionally active retrogenes like *EIF4A1P10* or *TCF3P* lost in some members of African populations, for example [34,35].

Numerous analyses have shown that the retroposition process was particularly intensive during the evolution of primates. The intensity of this phenomenon is associated with the occurrence of many retrocopies specific for this order of mammals [36,37]. As a result of this “burst of retroposition”, retropseudogenes belong to the largest group within all human pseudogenes [38]. Many retrogenes have been linked to cancer and a lot of them are human and primate-specific as it is demonstrated further down. Therefore, the question arises whether a large number of human retrogenes are associated with a high risk of neoplastic transformation in our species.

## 4. Cancer and Evolution

**Figure 3:**
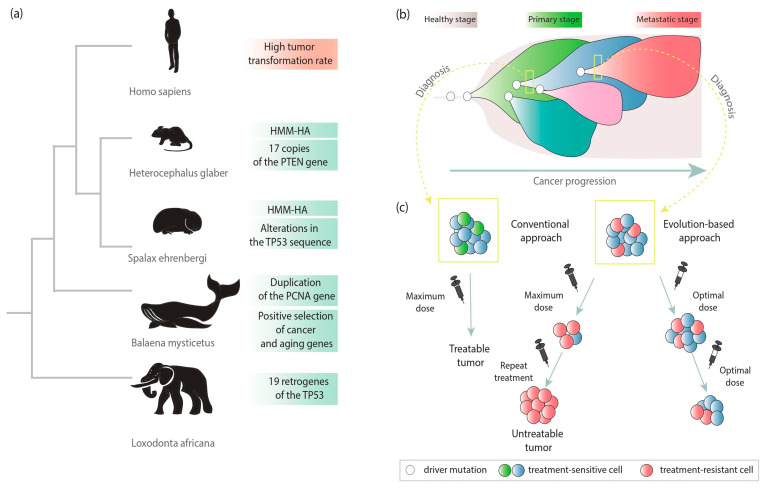
Evolutionary cancer mechanisms in the context of species-specific features in cancer suppression (**a**), clonal subpopulations within a tumor (**b**), and cancer treatment (**c**).

Mutational events form the basis of species evolution as well as cancer development [9]. Cancer tumors are highly dynamic and adaptive systems and evolve very quickly. Evolutionary processes play a role in the progression of cancer on two levels, at the level of species evolution and the level of individual cancer development (Figure 3). The phenomenon of natural selection operates on specific features of the population associated with cancer promotion/suppression [11]. Mechanisms of resistance to tumor transformation have been described in several species [39]. Cancer tumors are also subject to natural selection, and the “branched evolution” of species is reflected in the evolutionary trajectories of cancer cell populations [40].

### 4.1. Species-Specific Features of Cancer Suppression

The riddle “Peto’s paradox” indicates that the incidence of cancer among animals does not increase with body size and length of life. A good example constitutes already mentioned studies on a group of African elephants (lat. *Loxodonta africana*) and Asian elephants (lat. *Elephas maximus*). Among elephants, in comparison to other species, the tumor transformation rate is lower than expected. Unlike human cells, with one copy of the *TP53* gene, African elephants have 20 copies, 19 of which arose from retroposition [12]. The *TP53* gene encodes the p53 protein, which is called the “genome guardian” [41]. It belongs to key suppressor genes, and *TP53* mutations have been observed in most human cancers [42,43]. Disruption of p53 protein function causes the occurrence of cancer cell features [12]. The presence of extra copies results in an effective DNA damage response through the hyperactive *TP53* pathway (Figure 3a) [44].

A similar phenomenon has been observed in the population of the long-lived (approximately 200 years) bowhead whale (lat. *Balaena mysticetus*), although the resistance mechanism is not entirely clear. This has been linked to the positive selection force acting on cancer and aging genes involved in DNA damage repair and thermoregulation, *ERCC1* and *UCP1,* respectively. Duplication of the *PCNA* gene, one of the essential repair mechanism genes, has been reported. This can reduce the frequency of mutations and thus prevent tumorigenesis [45,46].

A species-specific cancer defense mechanism was also uncovered in the long-lived rodent lineage, including the naked mole-rat (lat. *Heterocephalus glaber*) [13]. The naked mole-rat resistance mechanism is based on the limitation of cell proliferation through the expression of high molecular mass hyaluronan (HMM-HA). The longer variant of HMM-HA inhibits divisions, inflammation, and metastatic processes [47]. It is quite interesting that in the naked mole-rat genome, 17 additional copies of *PTEN*, an important tumor suppressor gene, have been also reported. This may additionally contribute to such strong resistance to cancer [48]. Another example of a rodent that has evolved a way to suppress cancer is the blind mole-rat (lat. *Spalax ehrenbergi*). In this case, a subterranean lifestyle is connected to unusual tolerance to hypoxia. This has been associated with alterations in the *TP53* gene sequence. Similar changes have been identified in hypoxia-tolerant human tumors [13,49]. Interestingly, despite many studies, no cases of malignant neoplasm have been found in this species [44].

The literature data also report bats as a relatively long-lived species. It has been suggested that the ability to fly as an energy-intensive activity has caused the evolution of mechanisms that inhibit oxidative stress. Furthermore, DNA damage checkpoint genes are under positive selection in this case. These effects may be related to cancer resistance in the bat population [39].

### 4.2. Evolution within Cancer Tumors

The impact of evolutionary forces is also visible in cancer cells. It is supposed that a tumor basically consists of copies of a single cell. During tumor development, neoplastic changes (e.g., mutations) create heterogeneous masses—the starting point for the operation of evolutionary pressure. However, mutations are not the only forces shaping cancer evolution. Successive changes lead to the formation of different genetic subclones (Figure 3b) [11,40]. At this point, the evolutionary selection is also starting to play a role, and one group of tumor cells may be evolutionarily favored. This situation may occur when some cells develop traits that give them an advantage in a particular tumor environment. As a result, these cells will have more “offspring” than others. Furthermore, the high degree of tumor diversity and genomic instability results in a high risk of an adaptive mutation, which in turn is related to a faster progression of the disease [50].

Additional confirmation of the evolutionary forces acting within cancer comes from genetic changes in the response to a particular drug (Figure 3c). The diagnosed tumor may consist only of cells that are sensitive to treatment, and the patient has a good prognosis. However, among some fraction of patients, after applying certain therapies, the response is observed only at the initial phase and, unfortunately, tumor progression unexpectedly accelerates. This may indicate the presence of therapy-resistant subpopulations in the pretreated tumor. It has been also noted that therapy-derived selective pressure can determine the growth of therapy-resistant populations and induce the onset of “acquired resistance”. Therefore, adaptive therapy has been proposed, whereby maintaining a population of drug-sensitive cells limits the growth of populations resistant to treatment. The evolution-based approach relies on the combination of different drugs or their doses to slow tumor proliferation. It is suggested to use repeated optimal doses to reduce tumor volume rather than destroying it. This less aggressive approach may allow better control for the tumor and prevent the development or widespread of more aggressive, treatment-resistant form. This switch from the traditional approach that bases on maximal cell death to maximum progression-free survival could improve cancer treatment outcomes [40,51,52,53].

Just as the evolutionary history of a given species has led to differences in susceptibility to cancer, so does the history of tumor development influence the response to applied oncological treatment. Thus, determining the course of the evolutionary history of the tumor is important for establishing the best oncological treatment for a particular type of tumor and the stage of its development. The evolution of species-specific resistance mechanisms to cancer and the occurrence of specific tumor types and patient responses to treatment have been linked in some cases with retroposed genes as described below.

## 5. Retrogenes in Cancer

Identification of new biomarkers that will help predict a series of events in cancer evolution would certainly lead to more effective diagnostics and treatment. Retrocopies seem to be perfect candidates. The literature has shown a relationship between the expression level of some retrogenes and the occurrence of specific cancers [14,15]. Retrocopies involved in the response to a particular treatment, such as radiation [10] or paclitaxel [54], have also been reported [10,54]. Moreover, many studies describe retropseudogenes associated with the occurrence of a particular stage or form of the tumor [55,56,57]. It turns out that they can play multifaceted roles within tumor cells, and the literature reports retrogenes that are both oncogenes and tumor suppressors. A list of the cancer-related retrocopies that have been described so far in the literature is presented in Table 1.

### 5.1. Increased Expression in Cancer

Retrogenes with elevated expression in cancer constitute a large group, and in many cases, increased expression of these retrocopies promotes cancer development. The expression levels of retrocopy *KRASP1* are correlated with the prostate cancer phenotype. Its parental gene—*KRAS*—belongs to one of the most well-known oncogenes. Cancer cell line studies have shown that *KRASP1* overexpression causes increased parental gene expression and cell proliferation [59]. It has also been hypothesized that predisposition to ovarian cancer is associated with the expression of the small subunit processome component *UTP14C*, a protein-coding retrocopy of the *UTP14A* gene. This was explained by *UTP14C* downregulation of *TP53* levels, which leads to the prevention of cell cycle arrest and apoptosis [60]. Upregulated expression of *MSL3P1*, male-specific lethal-3 homolog pseudogene 1, has been correlated with renal cell carcinoma [61]. Other examples of retrocopies overexpressed in cancer tissues include *ANXA2P2* [62], *CSDAP1* [63], *LGMNP1* [64], *UBE2CP3* [65], *RACGAP1P* [66], *PTTG3P* [67], and *CKS1BP7* [68].

Analyses of RNA-seq data performed in our group revealed that more retrogenes may be associated with cancer. Differential expression analysis allowed the identification of 3 potential markers with increased expression levels characteristic of breast cancer, *RPL5P4*, *ASS1P2*, and *AC007731.2*, and 8 retrocopies with elevated expression in lung adenocarcinoma, *PTBP1P*, *AL121949.1*, *HNRNPA3P9*, *retro_hsap_4319*, *AC090695.2*, *CDK8P2*, *MSL3P1*, and *POLR3GP1* [89]. *Retro_hsap_4319* is a novel retrogene, i.e., not annotated in the reference genome, placed in the RetrogeneDB database [58].

### 5.2. Decreased Expression in Cancer

Retrogenes associated with tumor suppression have also been reported. Downregulation levels of *PTENP1* have been associated with gastric cancer and renal cell carcinoma. The *PTENP1* functions as a miRNA sponge. A decreased level of *PTENP1* contributes to increased degradation of its oncosuppressive parental gene, *PTEN,* which exerts a growth-inhibitory role within the tumor [15,59,69,70,71]. Another example is *INTS6P1* retrocopy. Its lower serum levels correspond to hepatocellular carcinoma. Interestingly, the diagnostic power of this retrocopy is comparable to the most common biomarker for hepatocellular carcinoma—α-fetoprotein [15,72]. In turn, the expression of *TUSC2P1* retrocopy suppresses the proliferation and migration of cancer cells and promotes apoptosis. This duplicate share sites for miRNAs with its progenitor *TUSC2* gene, thereby regulating its expression. The interaction with common miRNAs promotes parental gene expression and results in inhibition of proliferation, migration restriction, and apoptosis induction [73]. An additional example of a tumor suppressor is *NKPL*. Downregulation of this retrocopy is connected with lower overall survival in several cancers, including kidney renal papillary cell carcinoma, pancreatic adenocarcinoma, and adenoid cystic carcinoma [74]. A decreased expression level of *CTNNA1P1* has been associated with the pathogenesis of colorectal cancer. Suppressive action of the cognate gene *CTNNA1* has also been shown in several tumors [75]. An example of the well-described retrogene in the cancer literature is *RHOB* exhibiting suppression activity. Decreased expression of *RHOB* has been reported in many cancer studies [76,77,78,90].

The previously mentioned analysis of breast cancer samples led to the identification of 17 additional retrocopies with decreased expression levels (*AC104212.2*, *RHOQP2*, *NKAPL*, *RPL21P16*, *RBMS1P1*, *retro_hsap_2623*, *DIO3*, *FAM122A*, *RPSAP70*, *PTENP1*, *AC138392.1*, *DHFR2*, *CTB-50E14.5*, *AK4P1*, *RAB43P1*, *PSMA2P1*, and *RBMXL1*). In the lung cancer cohort, 13 retrogenes showed decreased expression in cancer (*RPL13AP17*, *HNRNPA1P33*, *SIRPAP1*, *AL136982.4*, *AL136452.1*, *AC084880.1*, *HMGN2P15*, *CDC20P1*, *AC022217.1*, *DIO3*, *HMGB3P10*, *BET1P1*, and *TMED10P2*) [89].

### 5.3. Subtype-Specific Retrogenes

Differences in the expression level of retrogenes were also observed depending on the subtype of cancer. Retrocopies of the *HMGA1* gene have been related to the occurrence of anaplastic thyroid carcinoma. *HMGA1P6* and *HMGA1P7* have oncogenic activity and contribute to cancer progression. In well-differentiated and weakly aggressive papillary thyroid carcinoma, *HMGA1P6* and *HMGA1P7* were not identified. In turn, anaplastic thyroid carcinoma, one of the most malignant cancers in humans, expresses high levels of these retrogenes [14]. Interestingly, a similar relationship has been noted among patients with endometrial cancer—increased expression levels of *HMGA1P6* and *HMGA1P7* correlate with the malignant phenotype [80]. Another good example is the upregulated *SUMO1P3* retrocopy in gastric cancer patients, which has the marker potential to differentiate between cancer and benign gastric disease [15,82].

In our laboratory breast cancer analysis, two retrocopies with differential expression characteristics for the ER+ (estrogen receptor-positive) subtype, *AC098591.2,* and *PABPC4L*, and 7 downregulated retrocopies in the TNBC (triple-negative breast cancer) subtype, *RAB6C, RPS16P5*, *RHOB*, *MEIS3P2*, *PGAM1P5*, *HMGN2P15*, and *KRT8P13*, were identified [89].

### 5.4. Stage-Specific Retrogenes

Increased expression of *NANOGP8* and *POU5F1P4/P5* retrogenes has been correlated with the phenotype of cancer stem cells (CSCs). The occurrence of this subpopulation, with high metastatic capacity, heralds intensive tumor expansion. In addition, the altered expression of a retrocopy of parental genes associated with maintaining pluripotency (*NANOG* and *POU5F1*) may also be a sign of early disease relapse [27,55]. It is worth noting that the knockdown of *NANOG* and *NANOGP8* reduces the malignant transformation in prostate cancer cells [85]. Another example of cancer stage-specific retrocopy is *SLC6A6P1*, also known as *SLC6A610P*, associated with recurrence in high-grade serous ovarian cancer. This subtype of ovarian cancer is very common (over 70% of affected women) [91]. Moreover, due to the lack of reliable diagnostics, it is usually detected at an advanced stage [87].

The expression of the *PDIA3P1* retrocopy was significantly increased in hepatocellular carcinoma. Interestingly, it has been demonstrated that the *PDIA3P1* expression level is related to metastasis and TNM stage and that a knockdown of retrocopy causes reduced migration and invasion of cancer cells [56]. One of the metastasis-related retrocopies is the previously described *CTNNA1P1*, whose expression has been significantly correlated with node metastasis in colorectal cancer patients [75]. Cooke et al., sequenced samples from different stages of lung and colon cancer. Their analysis revealed retrocopies unique for a given stage. Nevertheless, they have also found several processed pseudogenes that are expressed in both the primary tumor and metastasis [9].

### 5.5. Treatment Response-Related Retrogenes

Examples of retrogenes that are associated with the response to a particular treatment can also be found in the literature. The expression of the retrocopy *PPIAP43*, for instance, has been correlated with radiosensitivity in a patient with small-cell lung cancer [10]. This discovery is quite important since radiation constitutes the main strategy in the case of this cancer. The sensitivity to radiation differs among oncological patients, but to date, there is no suitable biomarker. Another example is the *FTH1P3* retrocopy, which promotes *ABCB1* expression by sponging miR-206. As a result, resistance to paclitaxel is activated in breast cancer patients [54].

The relationship between the sequence variant of a given retrocopy and the individual’s prognosis has also been described. The occurrence of the *E2F3P1* GA/AA allele at the rs9909601 locus has been associated with higher overall survival among hepatocellular carcinoma patients [15,88].

### 5.6. mRNA Sponging as the Main Retrocopy Mechanism in Cancer

A leading role of retrogenes described in the literature is sponging the miRNAs. This posttranscriptional process regulates parental or other genes when they share binding sites for miRNAs [6]. Recently, a genome-wide analysis demonstrated that as many as 181 retrocopies putatively regulate 250 transcripts of 187 genes [24].

**Figure 4:**
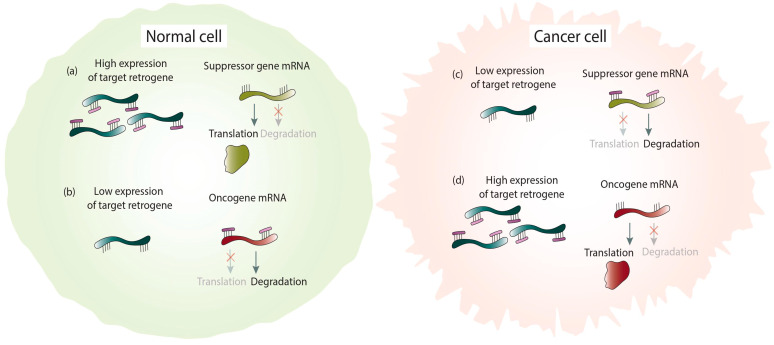
Cancer-related retrocopies as miRNA sponges in normal and cancer cells. Binding miRNAs to the highly expressed retrogene and translation of the suppressor gene (**a**). Degradation of the oncogene mRNA by miRNAs binding due to a low retrogene expression (**b**). Degradation of the suppressor gene mRNA by miRNAs binding because of low retrogene level (**c**). Binding miRNAs to the high expressed retrogene and translation of the oncogene (**d**).

Under normal conditions, there is a balance in the expression level of retrocopies. Sufficient expression of retrocopies regulates suppressor genes by competing for shared miRNAs. This prevents suppressor gene transcript degradation and enables the translation process (Figure 4a). Consequently, the low expression level of retrogenes contributes to increased miRNA binding to suppressor genes, which drives them on the path of degradation and promotes cancer transformation (Figure 4c). A good example represents the decreased expression of the *PTENP1* retrocopy in cancer [15].

The opposite is true in the case of oncogenes. In a normal cell, low expression of a retrogene that shares binding sites with oncogenes results in a lack of competition for common miRNAs. As a result, miRNAs bind to oncogene mRNAs and direct them on the degradation path (Figure 4b). Under cancer conditions, elevated expression of a given retrocopy causes sponging miRNAs and prevents oncogene degradation (Figure 4d), which leads to cancer development. This type of relationship occurs in the abovementioned *HMGA1* gene and its retrocopies [14].

## 6. Phylogeny of Cancer-Related Retrogenes

**Figure 5:**
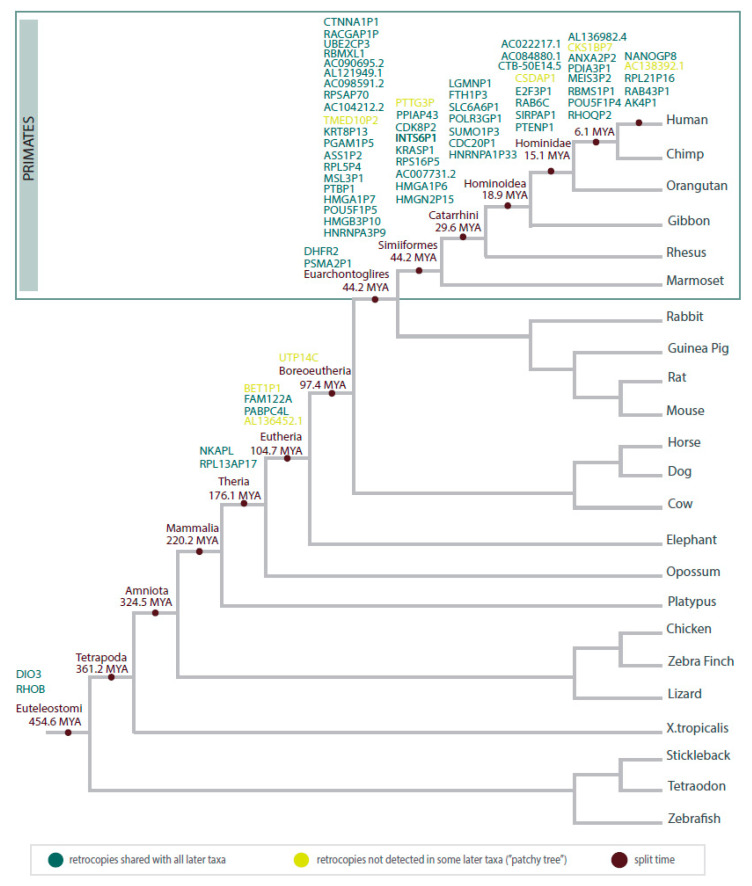
Schematic tree illustrating the estimated time of the origin of cancer-related retrocopies during animal evolution.

We used the GenTree database (http://gentree.ioz.ac.cn/) [92] to determine the time of cancer-related retrogene origination. Figure 5 represents the estimated point of the origin of earlier described retrogenes (no data for *TUSC2P1*, *retro_hsap_2623,* or *retro_hsap_4319*). Some of them are characterized by heterogeneous origins (“patchy tree”), but future research is needed to establish whether this results from independent retroposition events or loss of retrocopy in some species.

The oldest retrocopies recognized as cancer-related are *DIO3* and *RHOB*. Both arose during the early evolution of vertebrates and represent protein-coding retrogenes. They are widely distributed in animal genomes and well described in the literature. Conservation of ORFs in these quite old retrogenes may indicate that they acquired transcriptional capabilities very quickly and the propensity to accumulate mutations, typical for retrocopies, was “locked” due to the functional importance of gene products. *RHOB* is an important oncosuppressor, and a decrease in its expression promotes cancer, as described earlier. Increased expression of the *DIO3* retrocopy has been related to tumor progression in papillary thyroid cancer and colon cancer [93]. In turn, a decrease in the level of *DIO3* expression has been described in lung and breast cancer [89]. Old retrocopies, well recognized in human cancers and present in the genomes of all bony vertebrates, are great candidates for studies of the origins of neoplastic processes. The study of these genes may also be valuable for uncovering common features of tumors among species that are far away in the evolutionary tree. Nevertheless, a lack of data regarding species other than humans and mice seems to be the greatest difficulty in performing such research.

The majority of human cancer-related retrocopies are specific for primates. A large part of these groups arose before the split of New and Old-World Monkeys. There is also a group of retrocopies that are present in the human genome only. One of these is *NANOGP8,* the retrocopy of the *NANOG* gene that has 11 pseudogenes. Ten of them are derived from retroposition, and *NANOGP8* is evolutionarily the youngest. Interestingly, in the chimpanzee genome, all NANOG copies can be found, except *NANOGP8* [55]. Other cancer-related retrocopies unique to humans include *AK4P1*, *RAB43P1*, *RPL21P16*, and *AC138392.1*. Changes in the expression level in cancer have also been detected in the case of their parental genes, *AK4* in lung cancer [94], *RAB43* in gliomas [95], and *RPL21* in breast cancer [96].

The role of the newly arose genes is intriguing from an evolutionary point of view. It has been suggested that the presence of genes characteristic of a given lineage is related to phenotypic adaptation [97]. Furthermore, it was noted that the expression of some evolutionarily young genes occurs specifically or preferentially in tumors. These genes were termed tumor-specifically expressed, evolutionarily novel (TSEEN) [38,98]. Moreover, it has been reported that new genes are also overrepresented among the testis and brain. It has been hypothesized that new genes can be recruited to processes under strong selection pressure (e.g., spermatogenesis, immune response) or processes involving novel organ development (placenta, expanded brain) [92]. We searched the GTEx database to assess the expression levels of cancer-related retrocopies in human normal tissues (Table S1) [99]. The results of the GTEx analysis are in agreement with the abovementioned statements. In the analyzed cohort, there were 25 retrocopies with low or no expression levels in normal tissues (max median TPM < 1). These genes are good candidates for potential TSEEN genes. Additionally, eight neoplastic retrocopies are active mainly in testis and brain tissues: *CSDAP1*, *AC022217.1*, *SLC6A6P1*, *KRT8P13*, *MSL3P1*, *HMGA1P7*, *RACGAP1P*, and *NKAPL*.

As we stated in the previous chapters, the process of tumorigenesis is common across the evolutionary tree. The study of human-specific retrocopies and differences in the retrocopy repertoire across vertebrate species may be essential for better understanding the high rate of neoplastic processes in our species. Moreover, retrogenes included in the TSEEN group, due to their species and tissue specificity, represent great potential as new tumor biomarkers.

## 7. Conclusions

This review aims to gain insight into the link between the evolutionary origin of high cancer incidence among humans and protein-coding gene retroposition. Retrocopies are known evolutionary players in the context of species-specific traits and are also involved in the carcinogenesis process. It is possible that retrogenes, as important elements involved in the evolution of species, also count in the “microevolution” of cancer tumors. The studies discussed herein underline that investigation of retrogene expression can be a useful diagnostic tool, especially when conventional clinical methods are not sufficient. Nevertheless, retrocopies are often omitted as candidate genes and considered artifacts. Low interest in these sequences in cancer studies may also come from the fact that analysis of their expression is a significant challenge due to their low level compared to genes encoding proteins and high level of sequence similarity with their progenitors. However, the number of retrocopies identified thus far demonstrates that these gene duplicates are quite important actors in carcinogenesis.

Considering the expanding research in the field of evolutionary medicine, retrogene analysis seems to be a promising direction for future investigation. The studies summarized here open the way for using retrogene expression evaluation both to indicate cancer subtype/stage and to predict patient treatment responses. This may improve cancer diagnostics, customize more tailored therapy, and affect the prognosis of oncological patients. In addition, the evolutionary-based view of cancer can provide information about human-specific traits and the direction of our evolution.

## Figures and Tables

**Table 1:** Characteristics of the cancer-related retrogenes that have been described in the literature (based on [15,57]).

Retrocopy	Ensembl ID	RetrogeneDB ID [58]	Chromosome	Parental Gene	Cancer Type
*KRASP1*	ENSG00000220635	retro_hsap_3474	6	*KRAS*	prostate cancer [59]
*UTP14C*	ENSG00000253797	retro_hsap_29	13	*UTP14A*	ovarian cancer [60]
*MSL3P1*	ENSG00000224287	retro_hsap_2401	2	*MSL3*	renal cell carcinoma [61]
*ANXA2P2*	ENSG00000231991	retro_hsap_4150	9	*ANXA2*	hepatocellular carcinoma [62]
*CSDAP1 (YBX3P1)*	ENSG00000261614	retro_hsap_1674	16	*YBX3*	lung adenocarcinoma [63]
*LGMNP1*	ENSG00000214269	retro_hsap_1272	13	*LGMN*	glioblastoma [64]
*UBE2CP3*	ENSG00000250384	retro_hsap_2935	4	*UBE2C*	hepatocellular carcinoma [65]
*RACGAP1P*	ENSG00000257331	-	12	*RACGAP1*	hepatocellular carcinoma [66]
*PTTG3P*	ENSG00000213005	-	8	*PTTG1*	breast cancer [67]
*CKS1BP7*	ENSG00000254331	-	8	*CKS1B*	breast cancer [68]
*PTENP1*	ENSG00000237984	retro_hsap_4245	9	*PTEN*	hepatocellular carcinoma [69], gastric cancer [70], renal cell carcinoma [71]
*INTS6P1*	ENSG00000250492	retro_hsap_3307	5	*INTS6*	hepatocellular carcinoma [72]
*TUSC2P1*	ENSG00000285470	-	Y	*TUSC2*	esophageal squamous cell carcinoma [73]
*NKAPL*	ENSG00000189134	retro_hsap_15	6	*NKAP*	kidney renal papillary cell carcinoma, pancreatic adenocarcinoma, adenoid cystic carcinoma [74]
*CTNNA1P1*	ENSG00000249026	-	5	*CTNNA1*	colorectal cancer [75]
*RHOB*	ENSG00000143878	retro_hsap_108	2	*RHOA*	renal cell carcinoma [76], lung cancer [77], colorectal cancer [78]
*HMGA1P6*	ENSG00000233440	retro_hsap_1175	13	*HMGA1*	endometrial carcinoma [79], ovarian carcinosarcoma, thyroid carcinoma [14]
*HMGA1P7*	ENSG00000216753	-	6	*HMGA1*	endometrial carcinoma [79], ovarian carcinosarcoma, thyroid carcinoma [14], breast cancer [80]
*SUMO1P3*	ENSG00000235082	retro_hsap_240	1	*SUMO1*	hepatocellular carcinoma [81], gastric cancer [82], colorectal cancer [83]
*NANOGP8*	ENSG00000255192	retro_hsap_1549	15	*NANOG*	gastric cancer [84], prostate cancer [85]
*POU5F1P4 (OCT4-pg4)*	ENSG00000237872	-	1	*POU5F1*	hepatocellular carcinoma [27]
*POU5F1P5 (OCT4-pg5)*	ENSG00000236375	-	10	*POU5F1*	endometrial carcinoma [86]
*SLC6A6P1*	ENSG00000226818	retro_hsap_2498	21	*SLC6A6*	ovarian cancer [87]
*PDIA3P1*	ENSG00000180867	retro_hsap_217	1	*PDIA3*	multiple myeloma [56]
*PPIAP43*	ENSG00000255059	retro_hsap_816	11	*PPIA*	small cell lung cancer [10]
*FTH1P3*	ENSG00000213453	retro_hsap_2240	2	*FTH1*	breast cancer [54]
*E2F3P1*	ENSG00000267046	retro_hsap_1749	17	*E2F3*	hepatocellular carcinoma [88]

## Data Availability

Not applicable.

## Supplementary Materials

The following are available online at https://www.mdpi.com/2075-1729/11/1/72/s1, Table S1: GTex analysis.
